# Unraveling Tyrosine Hydroxylase in the Brain of a Crustacean: An Integrative Investigation Using Different Approaches

**DOI:** 10.1007/s12035-026-06269-x

**Published:** 2026-10-20

**Authors:** Carlos Augusto Borges de Andrade Gomes, Thyago R. Cardim-Pires, Rafaela Rocha dos Santos, Maria Clara da Costa Simas, Andressa de Abreu Mello, Louise Caroline Vitorino, Paula Grazielle Chaves da Silva, Debora Foguel, Clynton Lourenço Correa, Rosane Silva, Silvana Allodi

**Affiliations:** 1 Instituto de Biofísica Carlos Chagas Filho, Universidade Federal do Rio de Janeiro, Rio de Janeiro, RJ 21941-902 Brazil; 2 Instituto de Bioquímica Médica Leopoldo de Meis, Universidade Federal do Rio de Janeiro, Rio de Janeiro, RJ 21941-902 Brazil; 3 Centre de Recherche du CHU de Québec, Axe Neurosciences, 2705, Boulevard Laurier, Québec, QC G1V 4G2 Canada; 4 Center for Integration of Science and Industry, Bentley University, Waltham, MA 02452 USA; 5 Faculdade de Fisioterapia, Universidade Federal do Rio de Janeiro, Rio de Janeiro, RJ 22290-140 Brazil; 6 Programa de Pós-Graduação em Ciências Biológicas (Biofísica), Instituto de Biofísica Carlos Chagas Filho, Universidade Federal do Rio de Janeiro, Rio de Janeiro, 21949-902 Brazil

**Keywords:** Invertebrate neuroscience, Neurodegeneration, Catecholamines, Immunohistochemistry, Immunoblotting, Tyrosine hydroxylase expression

## Abstract

Tyrosine hydroxylase (TH), the rate-limiting enzyme in dopamine (DA) synthesis, is conserved across most animal phyla. DA regulates motor activity, sensory processing motivation, neural homeostasis, and protection against oxidative stress. TH and DA have been described in vertebrates and in some invertebrates, including arthropods. This study investigated the anatomical location of TH and DA and characterized the biochemical and molecular structure of TH in the brain of the mangrove crab *Ucides cordatus.* This species is a useful environmental model for neurodegeneration studies, due to its occurrence in metal-contaminated mangrove ecosystems, and as prey of other species, including humans. Using immunohistochemistry, immunoblotting, and structural modeling, we mapped TH presence and distribution in the crustacean brain and analyzed its molecular and evolutionary characteristics. Our results revealed a TH fragment structurally similar to fragments found in 21 other species and in a complex dopaminergic network in *U. cordatus*, characterized by diverse neuronal morphologies, widespread projections, and sensitivity to dopaminergic neurotoxins. These findings may support further studies on catecholaminergic neurobiology.

**Graphical Abstract:**

**Figure:**
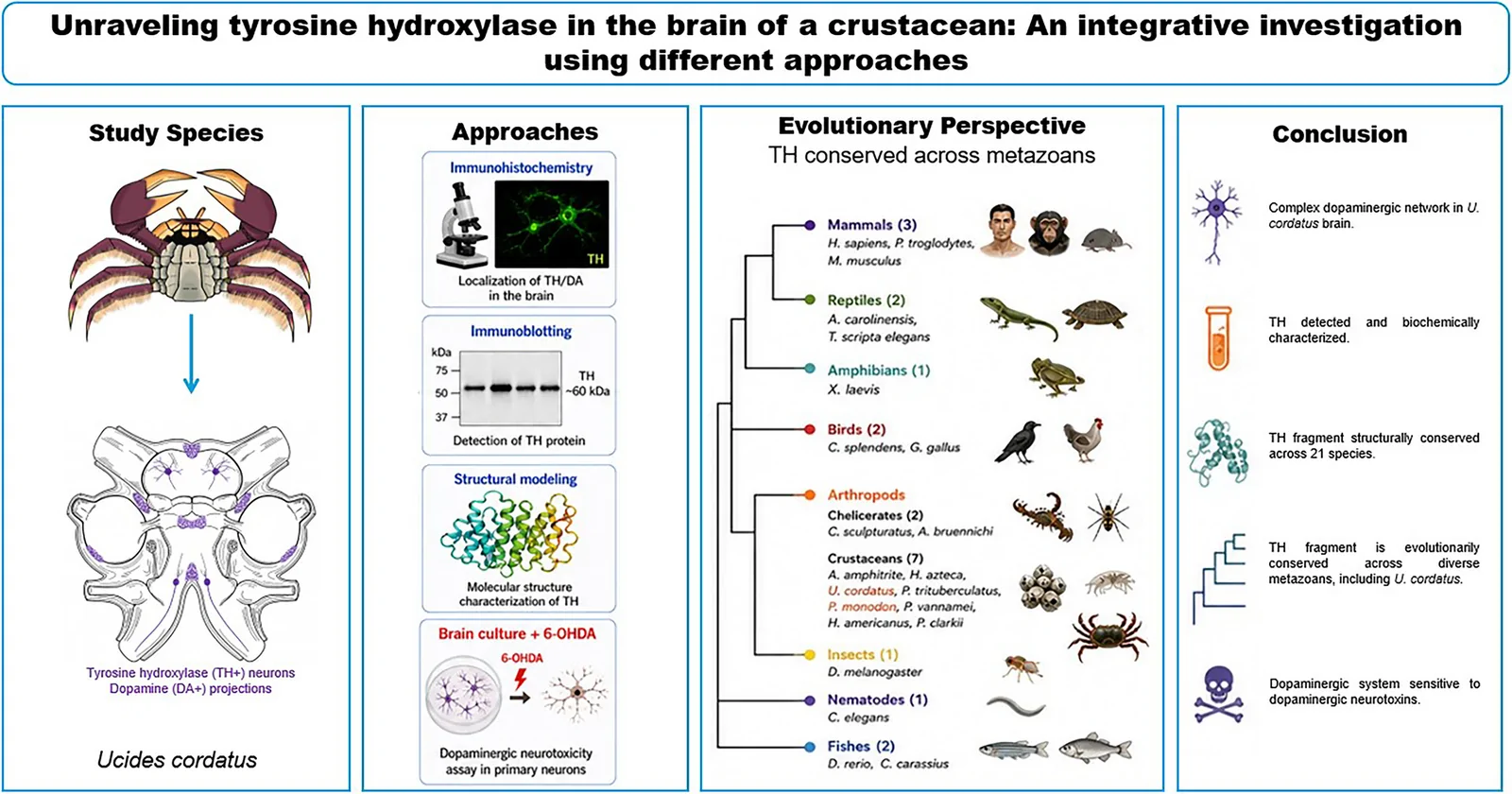

**Supplementary Information:**

The online version contains supplementary material available at https://doi.org/10.1007/s12035-026-06269-x.

## Introduction

Several morphophysiological changes in human neural cells are associated with dopaminergic neurons. These neurons are characterized by the presence of tyrosine hydroxylase (TH), the rate-limiting enzyme in dopamine synthesis [1–3]. In general, dopamine (DA) has roles in the reward system, motor and mood control, learning and memory, sleep processing, and lactation [4]. Evolutionarily, DA first appeared in the phylum Cnidaria and is present in most animal phyla [5].

Although DA production contributes to an oxidative environment, catecholamines may also function as scavengers of the free radicals generated during this process [4]. Thus, maintaining appropriate DA levels is essential for central nervous system (CNS) homeostasis and for limiting oxidative stress associated with the accumulation of reactive oxygen species (ROS) [6].

In humans, TH has four described isoforms due to alternative splicing of its mRNA [7]. These isoforms differ in expression levels and in the number of amino acids within their regulatory domains [8–10]. The anatomical locations of TH in vertebrates are diffused, consistent with its functions in the CNS and peripheral nervous system (PNS) [11, 12].

The isoforms of TH are called hTH1–4. Isoforms hTH1 and hTH2 are most often studied due to their occurrence throughout the vertebrate CNS and their evolutionary conservation [13]. In 2016, Bezem and collaborators [10], using crystallography techniques, revealed the complete and stable structure of isoforms hTH1 and hTH2. The C-terminal domain is composed of 40–50 amino acids that are related to the tridimensional folding of the enzyme [9]. The region with the active site has 330 amino acids and interacts with the substrate to regulate enzyme activity [13, 14].

In invertebrates, the dopaminergic phenotype is found in phyla with more plesiomorphic neural features, such as cnidarians. Physiological studies have shown that DA inhibits spawning in corals as well as in more recently derived phyla, including mollusks, echinoderms, and arthropods [5]. Recently, DA in sponges has been found to regulate cellular plasticity through both transcriptional and posttranslational mechanisms [15]. Dopaminergic neurons have also been described in annelids, in which exposure to neurotoxic agents such as rotenone induces effects comparable to those observed in vertebrate models of neurodegeneration [16]. Moreover, total ablation of the cerebral ganglion in the annelid *Eisenia fetida* stimulates dopaminergic signaling in the ventral nerve cords, promoting complete tissue regeneration [17].

In the CNS of crustaceans, DA, and consequently TH, have been found in the optic stalks and cerebral ganglia of the crabs *Scylla olivacea* and *Chasmagnathus granulatus* (now called *Neohelice granulata*) and the crayfish *Orconectes rusticus* [18–20]. These molecules have also been found in the ventral nerve cords [20] and stomatogastric ganglia [21, 22] of *C. granulatus* and the lobster *Panulirus interruptus*.

In our study model, the swamp ghost crab *Ucides cordatus*, neither this enzyme nor DA has yet been described. Therefore, characterization of these molecules may broaden investigations under physiological conditions and models of neurodegeneration. This crab inhabits mangrove ecosystems, which often contain high concentrations of heavy metals such as iron and manganese. Heavy metals are associated with dopaminergic degeneration and parkinsonism and may be biomagnified in the food chain, making this species a potentially valuable environmental model for studying nervous system degeneration [23], similarly to the use of the crustacean *Daphnia pulex* as a model to investigate Parkinson’s disease (PD) mechanisms [24].

**Fig. 1:**
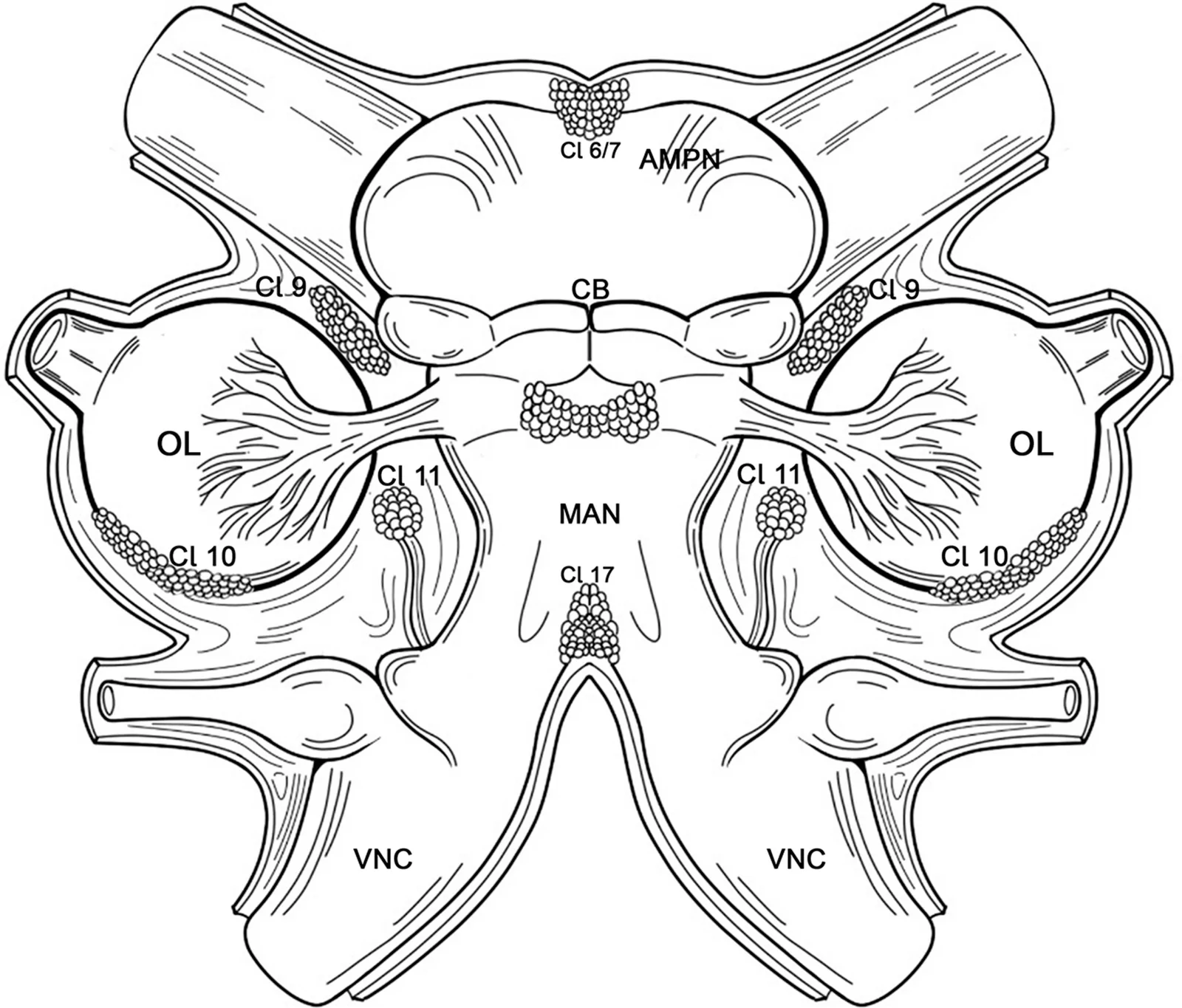
Scheme of the brain areas of the crab *Ucides cordatus* (protocerebrum, deutocerebrum, and tritocerebrum). Cl 6/7 = clusters 6 and 7; AMPN = anterior medial protocerebral neuropils; Cl 9 = cluster 9; CB = central body; OL = olfactory lobe; Cl 11 = cluster 11; MAN = medial antenna I neuropil; Cl 10 = cluster 10; Cl 17 = cluster 17; VNC = ventral nerve cords

The overall structural organization of the cerebral ganglion (brain) of the crab *U. cordatus* is composed of the protocerebrum, deutocerebrum, and tritocerebrum. The protocerebrum is divided into a lateral and a median protocerebrum. Enclosed within the median protocerebrum are the protocerebral bridge (PB) and the central body (CB), which are composed of cells and fibers [25, 26]. The deutocerebrum consists of the olfactory lobe (OL), a paired structure constituted by projection neurons located in two cell clusters, cluster 9 (Cl 9) and cluster 10 (Cl 10). These clusters send projections to another group of cell bodies, cluster 11 (Cl 11) [25, 27]. Another group of cell bodies, cluster 6 (Cl 6), is constituted by neurons that project to the ventral region, related to motor functions [18]. The tritocerebrum, the last division of the crustacean brain, contains cluster 17 (Cl 17) that sends projections to the ventral nerve cords, which control digestive and motor regions [28]. The present study also revealed the connection between Cl 6 and Cl 17 through the anterior medial protocerebral neuropils (AMPN) and the medial antenna I neuropils (MAN), located medially to the OL [25] (Fig. 1).

TH is the rate-limiting enzyme in catecholamine biosynthesis [9], and its structure has been well characterized in vertebrates. In the present study, we investigated TH in *U. cordatus* by (1) identifying TH- and DA-positive regions in the brain using immunohistochemistry; (2) comparing the TH molecular weight with that of vertebrate homologs by immunoblotting; (3) identifying a TH fragment through RNA sequencing, followed by sequence alignment and tertiary structure prediction using AlphaFold; and (4) performing a phylogenetic analysis to assess the evolutionary relationships of TH.

## Materials and Methods

### Animals and Ethical Committee

Two hundred and fifty-five adult males of *U. cordatus* (carapace width 5.9–8.3 cm), in the intermolt phase, were collected from mangroves in Itambi, Itaboraí, RJ, Brazil (S–22°43′59.99″, W–42°58′00.00″) and acclimated for 7 days in aquaria. The crabs were maintained under controlled conditions (water salinity 20 ppm, 25–28 °C, and 12L:12D) and fed leaves of the black mangrove *Avicennia schaueriana*. All procedures adopted in this study, including the location where the animals were captured, were conducted under a permit from the Brazilian Authority for the Use of Experimental Animals, Instituto Chico Mendes de Conservação da Biodiversidade (ICMBio), Ministério do Meio Ambiente (Certificate # 72335–1/ICMBio/2020).

### Immunohistochemistry

#### TH

Five animals were cryoanesthetized and dissected in 0.1 M phosphate-buffered crustacean saline (crust-PBS), pH 7.4, prepared according to [29], and composed of 205 mM NaCl, 5.4 mM KCl, 34.4 mM CaCl_2_, 1.2 mM MgCl_2_, and 2.4 mM NaHCO_3_. The brains were fixed in 2% paraformaldehyde and processed for Paraplast® embedding. Dewaxed paraffin Sects. (5 μm thick) of the brain were incubated for 15 min in methanol and 30% hydrogen peroxide, to expose all the binding epitopes. Next, the samples were rinsed with 0.1 M phosphate-buffer + Triton 0.5%, pH 7.4, for 30 min. Then, the slides were incubated in blocking solution with 10% goat serum (NGS) and 0.1 M phosphate buffer + Triton 0.3% for 15 min, followed by the primary antibody, the monoclonal rabbit anti-TH (Millipore, AB152; 1:50 dilution) at 4 °C for 2 days. Negative controls for the reaction were prepared by omitting the primary antibody.

At the end of this period, the slides were rinsed with phosphate buffer pH 7.4 + 0.5% Triton and incubated with the secondary antibody, the anti-rabbit Alexa Fluor® 546 (Invitrogen™ A11003, 1:600 dilution) for 2 h 30 min at room temperature. Next, the slides were rinsed twice with phosphate buffer pH 7.4 and incubated for 10 min with 4′,6-diamidino-2-phenylindole (DAPI; 1:10,000 dilution) to stain the cell nuclei. Fluoromount™ (Sigma Aldrich, F4680) mounting medium was added and the images were captured with a confocal microscope (Leica, SP8). All images were acquired with the same exposure times and adjusted for brightness and contrast, in accordance with the manufacturer’s instructions.

#### DA

After fixation with 4% paraformaldehyde for 1 h, the brains of another five crabs (*n* = 5) were washed with crust-PBS and blocked for 1 h in a solution containing crust-PBS pH 7.4 + 0.3% Triton + 3% NGS (Millipore). Next, the brain samples were incubated with polyclonal rabbit anti-DA (Enzo, BMLDA1140-0100; diluted 1:100 in the blocking solution) for 2 days, at 4 °C. Negative controls for the reaction were prepared by omitting the primary antibody. Next, the ganglia were washed in crust-PBS for 30 min and incubated with the anti-rabbit Alexa Fluor® 546 (Invitrogen™ A11003, 1:600 dilution) for 2 h 30 min at room temperature. The material was then washed with crust-PBS pH 7.4 for 40 min and incubated for 20 min with DAPI (1:10,000 dilution) to stain the cell nuclei. Finally, the samples were again washed with crust-PBS pH 7.4 for 40 min and mounted with n-propyl gallate as whole mounts.

### Dot Blot

A pool of 15 crabs per experiment (to achieve *n* = 5, total = 60 ganglia) was dissected on ice after cryoanesthesia for 30 min. The samples were immersed in five cryotubes (each with 15 ganglia) in a cocktail of P2714-1BTL protease inhibitors (Sigma Aldrich) (diluted 1:10 in deionized water), along with phosphate buffer (Sigma Aldrich, 10 μL), phenylmethylsulfonyl fluoride (PMSF) (17.5 μL/mL), Laemmli buffer, and beta-mercaptoethanol (150 μL/mL). The same procedure was used to prepare a rat brain (Wistar male, 2 months old, weight 250 g, and kept in a cage with Granja RG brand wood shavings) as a positive control. BSA (Inlab) was used as a negative control. The samples were quantified using the Folin–Lowry method [30]. They were then pipetted onto a nitrocellulose membrane coupled to a film and covered with a minifold (Schleicher & Schuell, Inc., 03431) connected to a vacuum pump (Prismatec) for transfer to the membrane. The transfer took 1 min. The membrane was then washed once with TBS 1 × pH 7.6 and subsequently washed with TBS Tween 0.1% pH 7.6. Then, the samples were blocked with 5% nonfat milk for 1 h and incubated with the primary antibody, rabbit anti-TH (Merck/Millipore, AB152), diluted 1:2000 in TBS Tween 0.1%, overnight at 4 °C.

The samples were then washed with TBS Tween 0.1% pH 7.6 for 30 min and incubated with biotinylated rabbit secondary antibody for 1 h, diluted 1:1000 in TBS 1× pH 7.6. The developer used was biotin (Universal LSAB™ Kit-HRP Rabbit/Mouse/Goat). Afterward, the membranes were washed for 15 min with TBS Tween 0.1% pH 7.6 and incubated with avidin, diluted 1:1000 in TBS 1 × pH 7.6, for 1 h. The avidin used was Universal LSAB™ Kit-HRP Rabbit/Mouse/Goat. The membranes were then washed with TBS 1 × pH 7.6 for 30 min and immersed in a background removal solution (14.61 g of NaCl plus 1 g of SDS diluted in 500 mL of TBS 1 × pH 7.6) for 30 min and then washed for 30 min with TBS 1 × pH 7.6. The membranes were developed using the ImageQuant™ LAS 4000 at the Immunoassay Platform (PIA) of the Institute of Biophysics Carlos Chagas Filho, at the Federal University of Rio de Janeiro (UFRJ).

### Western Blotting

Four pools of 15 crabs (the same used for the dot blot) each were dissected on ice following cryoanesthesia, and brain samples were homogenized in 2-mL cryotubes (Ciencor SPL) containing a cocktail of protease inhibitors P2714-1BTL (Sigma Aldrich; diluted 1:10 in deionized water) + phosphatase buffer + phenylmethylsulfonyl fluoride (17.5 μL/mL) + Laemmli buffer + beta-mercaptoethanol (150 μL/mL). The samples were then sonicated and stored in a freezer at −80 °C. In addition to the crabs, a rat brain was dissected and immersed in the same cocktail of inhibitors mentioned above as a positive control. The protein concentration was determined according to the Folin–Lowry method [30]. The samples were loaded into a running gel using Bio-Rad equipment for 40 min at 60 mA. After the run, the gel was transferred in semidry mode for 1 h to a nitrocellulose membrane. After transfer, the membrane was washed for 30 min in Tris-buffered saline (TBS) + 0.1% Tween, pH 7.6, followed by blocking in 5% milk for 1 h. After, the membranes were incubated with the rabbit anti-TH (Merck/Millipore AB152) diluted 1:2000 in TBS Tween 0.1% overnight, at 4 °C.

After incubation, the membranes were washed in TBS and 0.1% Tween, pH 7.6 and incubated with a biotinylated secondary antibody against rabbit IgG for 1 h at 1:1000 dilution in TBS 1 × pH 7.6. The developer used was biotin (Universal LSAB™ Kit-HRP Rabbit/Mouse/Goat). The membranes were then washed with TBS + 0.1% Tween, pH 7.6, followed by incubation with avidin diluted 1:1000 in TBS 1 × pH 7.6 for 1 h. The avidin used was Universal LSAB™ Kit-HRP Rabbit/Mouse/Goat. At the end of the reaction, the membranes were washed with TBS 1 × pH 7.6, and finally, the membranes were developed at the Immunoassay Platform (PIA) of the Institute of Biophysics Carlos Chagas Filho, UFRJ, using the ImageQuant™ LAS 4000 biomolecular imaging device.

### Adult Primary Cell Culture

The procedure followed the steps described in [31]. Twenty crabs were used on each experiment day (1, 3, and 5) in each group (control, vehicle, and experiment) (*n* = 3 of 20 crab brains each day, totaling *n* = 180 crab brains). We used pools of 20 crab brains because the crab brain does not have many cells. With fewer than 20 crab brains, we reasoned that we would not have had enough cells for the statistical analyses. After dissection, the brains were placed in a Petri dish with Leibovitz’s L-15 medium with L-glutamine (0.3 g/L, Sigma Aldrich, L4386) supplemented with penicillin and streptomycin (100 µg/mL, Sigma Aldrich, P4333), Anforicin B® (5 µg/mL, Cristália, São Paulo, SP, Brazil), and 10% fetal bovine serum (FBS; 10%, Cultlab, Campinas, SP, Brazil). The pH was set at 7.4. The medium was reconstituted at a double concentration (2 ×) with ddH_2_O, and filtered on 0.22-µm cellulose membranes. After mechanical dissociation of the brains with a pipette, we conducted a chemical dissociation with 500 µL of TrypLE™ Express (Gibco, Life Technologies) at 37 °C for 5 min. Then, the TrypLE™ solution was removed and replaced with L-15 medium with FBS, antibiotics, and Anforicin B (5 mg/mL) with the same volume used for TrypLE™. After, we again dissociated the tissue with a pipette. Next, we added 500 µL of the supplemented L-15 medium and centrifuged the sample for 5 min at 1800 × *g.* We removed the resulting pellet, added 2 mL of the L-15 medium, and again dissociated the pellet.

Finally, we added 10 mL of supplemented L-15 medium and placed the cells on glass coverslips (15 mm, Glasscyto, Meclab, Jacareí, SP, Brazil) coated with poly-L-ornithine (Sigma Aldrich, P3655). The coverslips were then placed on a 24-well plate (Costar), 500 µL per well, in an oven set at 28 °C for three days, with no CO_2_. Twelve wells were used for each experiment day for each experiment group and were treated with 6-hydroxydopamine (6-OHDA, 100 µM diluted in the culture medium with 0.02% ascorbic acid, Sigma Aldrich). The remaining 12 wells were used as follows: control (6 wells) and vehicle group (group containing vitamin C without 6-OHDA diluted in the culture medium; another 6 wells). The wells with vitamin C and 6-OHDA were not exposed to light. After the incubation periods, we conducted the immunocytochemical reactions as described above in the Immunohistochemistry section. The images were captured with a confocal microscope (Leica, SP8). Quantification of TH+ cells was conducted after the capture of 30 pictures of each coverslip, forming a mosaic at 40× magnification, and was performed blinded. All cells of the resulting mosaics were counted following three criteria: 1. The cells must be clearly labeled.. 2. The nuclei must be visible. 3. All cells must be contained within the mosaic.

Statistical analyses were performed using GraphPad Prism, version 11.00 for MAC (GraphPad Software, La Jolla, CA, USA). The experiment comprised three independent biological replicates (*n* = 3 per group). Values from replicate wells belonging to the same experimental group were averaged within each independent culture, producing one value per biological replicate for statistical analysis. Data normality was assessed using the Shapiro–Wilk test. When the assumptions of normality and homogeneity of variance were met, differences among groups were analyzed using one-way ANOVA followed by Bonferroni multiple-comparisons test, with each 6-OHDA-administered group compared with both the control and vehicle groups. Data are presented as mean ± [SEM], and differences were considered statistically significant at *p* < 0.05.

### RNA Extraction

Five animals (*n* = 5) had their brains dissected and immersed in a TRIzol® solution (Invitrogen, 15596026) for 24 h and then homogenized in the same solution. Next, the samples were incubated for 3 min in a TRIzol® solution with chloroform (Merck) in a 0.2:1 mL ratio. Then, they were centrifuged (Sorvall® RC-5B Refrigerated Superspeed Centrifuge, DuPoint Instruments) for 15 min at 12,000 × *g*, at 4 °C. The supernatant was transferred to another tube.

Next, the samples were immersed in 250 μL of isopropanol (Merck) for 10 min, followed by centrifugation at 12,000 × *g*, at 4 °C, for 10 min. Then, the sample pellets were resuspended in 500 μL of ethanol (VETEC), vortexed, and centrifuged for 5 min at 7500 × *g*, at 4 °C. The pellet was resuspended in 50 μL of MilliQ water and placed in a water bath (55 °C) for 15 min, and the amount of RNA was measured using the Qubit RNA HighSensitivity Assay Kit (Thermo Fisher, Q32852) in the Qubit® fluorimeter (Thermo Fisher).

### cDNA Preparation

cDNA samples were prepared from the extracted RNA, with an input of 752 ng of RNA. cDNA was obtained using the SuperScript™ IV VILO™ MasterMix kit (Invitrogen 11756050) according to the manufacturer’s instructions. The reaction occurred in the thermocycler (Veriti™, Applied Biosystems), programmed as follows: 1. Annealing at 25 °C for 10 min. 2. Reverse transcription at 50 °C for 10 min. 3. Enzyme inactivation at 85 °C for 5 min.

### Amplification of TH

Next, 2.5 μL of cDNA was mixed with colorless 1× buffer, MgCl_2_ (1.5 mM), deoxyribonucleotide phosphate (dNTP, 0.2 mM of each dNTP pool), GoTaq® DNA polymerase enzyme (1.25 μ/μL for 2.5 μL of cDNA) (Promega), MilliQ water, and forward and reverse primers at a concentration of 0.4 mM. The primers used were as follows:

- PenaeusTH-2R (5′-CGCGCGACGAGTGGTGGCGGATG-3′)—(IDT–Integrated DNA Technologies, 299,049,844).
- TH-2F (5′-CGCGAGGTGTGGGACGTCCTCAAG-3′)—(IDT–Integrated DNA Technologies, 283,522,843).

The PCR reaction occurred in the thermocycler, programmed as follows: 1. Activation of GoTaQ enzyme at 95 °C for 2 min. 2. Annealing and extension in a 35 × repeat cycle (95 °C for 30 s, 55 °C for 30 s, and 72 °C for 1 min). 3. Final extension at 72 °C for 5 min.

After, 10 µL of each amplicon was mixed with 2 µL of loading dye (6X DNA Loading Dye, Thermo Fisher) and 10 µL of DNA ladder 100 bp (Invitrogen, 15628019) and run on a 2% agarose gel, developed by ethidium bromide in a UV Transilluminator (Bio-Imaging Systems, MiniBis Pro, Biomerica, Irvine, CA, USA).

### Genetic Sequencing and Alignment

The PCR products were sequenced by the Sanger method using the Bigdye™ terminator v3.1 cycle sequencing kit (Applied Biosystems, Foster City, CA, USA) according to the manufacturer’s instructions, using the same primers as above for independent reactions, at a concentration of 3.2 μM. The sequencing products were run in the 3500 ABI Genetic Analyzer (Applied Biosystems, Foster City, CA, USA). The sequencing data were analyzed using Geneious Prime (version 2026.0.2; www.geneious.com) to generate a consensus sequence from the forward and reverse sequencing data. The DNA consensus sequence was translated into protein in silico according to the standard genetic code. The resulting protein fragment was submitted as a query to Protein BLAST analysis at NCBI to search for similarity in other organisms. All these sequences were retrieved from GenBank and aligned using Geneious Prime (version 2026.0.2) with global alignment, free of gaps, using the Blosum62 cost matrix.

### Phylogenetic Tree

To construct the phylogenetic tree, 21 species were chosen, based on three criteria: (1) their use in genetic studies, including research on PD; (2) their phylogenetic proximity to the study model, *U. cordatus*; and (3) the availability of previously sequenced TH genes. The selected species were as follows: *Homo sapiens* (access number KAI4069453.1), *Pan troglodytes* (chimpanzee; access number XP_016775674.1), *Anolis carolinensis* (green anole; access number XP_062823714.1), *Trachemys scripta elegans* (red-eared slider turtle; access number XP_034626169.1), *Mus musculus* (house mouse; access number AAA40434.1), *Xenopus laevis* (African clawed frog; access number AAR23931.1), *Corvus splendens* (house crow; access number QOW95819.1), *Gallus gallus* (chicken; access number CAB62388.1), *Centruroides sculpturatus* (bark scorpion; access number XP_023240076.1), *Argiope bruennichi* (wasp spider; access number XP_055935929.1), *Amphibalanus amphitrite* (striped barnacle; access number XP_043230680.1), *Hyalella azteca* (amphipod; access number XP_018020964.1), *Drosophila melanogaster* (fruit fly; access number: CAA53802.1), *Caenorhabditis elegans* (nematode; access number CDG24121.1), *Portunus trituberculatus* (Asian blue crab; access number XP_045138592.1), *Penaeus monodon* (giant tiger prawn, access number XP_037788831.1), *Penaeus vannamei* (whiteleg shrimp; access number ANA78296.1), *Homarus americanus* (American lobster; access number XP_042215467.1), *Procambarus clarkii* (red swamp crayfish; access number XP_045604547.1), *Carassius carassius* (crucian carp; access number XP_059402605.1), and *Danio rerio* (zebrafish; access number AAI63639.1).

Phylogenetic analysis was performed using partial TH sequences from 22 species, including the 21 species listed above and the partial TH sequence obtained in the present study from *U. cordatus*. Genetic distances were calculated using the Jukes–Cantor model, and a phylogenetic tree was constructed using the neighbor-joining method. The reliability of the inferred phylogenetic relationships was assessed by bootstrap analysis with 1000 replicates. Bootstrap support values ≥ 50% were displayed on the tree.

To clarify, species were included in the sequence comparison and phylogenetic analyses for several reasons: *Drosophila*, *C. elegans*, zebrafish *Danio rerio*, and *Xenopus* as there are many genetic studies on these species; *Homo sapiens* was included to compare *U. cordatus* with our species; and chimpanzee (*Pan troglodytes*) was chosen due to the high homology with *Homo sapiens*. We chose other arthropods because they belong to the same phylum as the model of this study, *U. cordatus*. Within arthropods there were several crustaceans such as *Penaeus vannamei* (whiteleg shrimp), *Homarus americanus* (American lobster), and *Procambarus clarkii* (red swamp crayfish). The fish *Carassius carassius* (crucian carp) was used to compare with zebrafish. Other vertebrates, reptiles and birds, were chosen to check for homology (they are sister groups).

### Tertiary Structure of TH

After partial sequencing of TH mRNA from *U. cordatus*, the corresponding amino acid sequence was obtained by translation. The primary sequence of the region so identified was first aligned with TH sequences from *Homo sapiens*, *Homarus americanus*, and *Drosophila melanogaster*, using the UniProt alignment tool to assess sequence conservation. The translated sequence was then submitted to AlphaFold3 [32] for prediction of its tertiary structure. The resulting structural model was analyzed and compared with the human TH structure (PDB: 2XSN), used as a reference template. Structural comparisons were performed by superimposing the predicted *U. cordatus* peptide onto both the tetrameric and monomeric forms of the human enzyme.

## Results

### Anatomical Distribution of TH in the Crab Brain

**Fig. 2:**
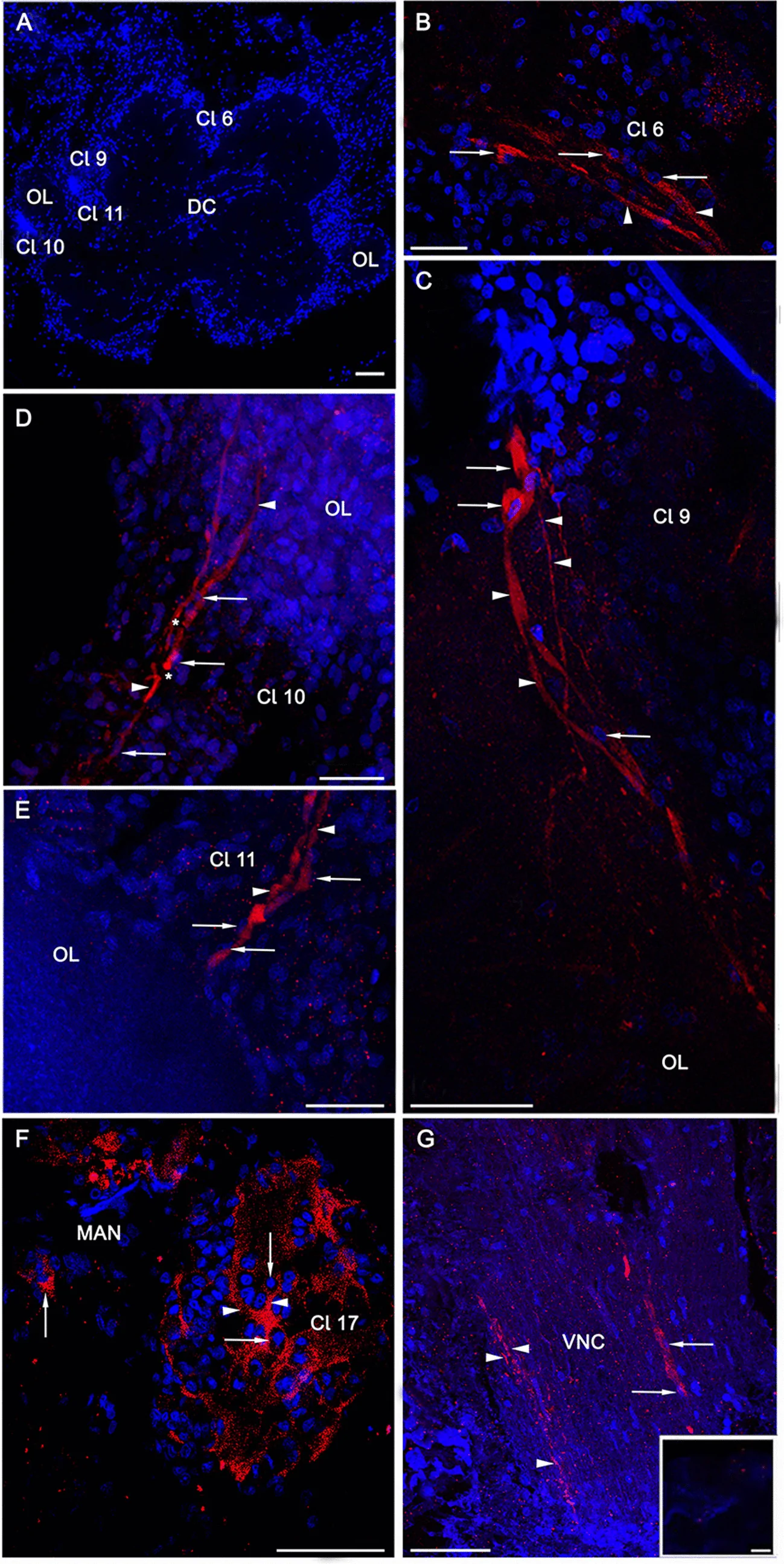
Immunohistochemistry for tyrosine hydroxylase (TH) in the brain of the crab *Ucides cordatus* (*n* = 5). **A** DAPI nuclear staining of the protocerebrum and deutocerebrum, showing the following regions: clusters 6, 9, 10, and 11 (Cl 6, Cl 9, Cl 10, and Cl 11); OL = olfactory lobe; DC = deutocerebrum commissure. **B** TH+ cell bodies (arrows) and their processes (arrowheads) within Cl 6. **C** Cell bodies (arrows) and processes (arrowheads) from Cl 9 to the interior of the OL. **D** Photomontage of confocal z-stack images (14 sections, 1 µm each) showing cell bodies (arrows) and processes (arrowheads) within Cl 10 projecting toward the OL. Note two gaps between two cell fibers (asterisks). **E** Cell bodies (arrows) and their processes (arrowheads) linking Cl 11 and the interior of the OL. **F** TH+ cell bodies (arrows) and processes (arrowheads) within cluster 17 (Cl 17) and medial anterior neuropil (MAN). **G** TH+ cell bodies (arrows) and processes (arrowheads) running in the ventral nerve cord (VNC). The insert shows a negative control of the reaction. Scale bars: **A** (200 µm), **B**–**G** and insert (60 µm)

The brain regions bearing TH in the crab were mapped using immunohistochemistry; the anatomical regions analyzed are shown in Fig. 2A. Cells with TH+ projections within Cl 6 and Cl 9, with dimensions of large neurons and projections of different widths, are shown in Fig. 2B and C. Figure 2D displays a confocal photomontage (z-stack; 14 sections at 1 µm each) revealing at least two processes originating within Cl 10 and projecting toward the OL. Figure 2E shows a few TH+ cells extending toward OL and projecting elsewhere. The immunohistochemical reaction for TH also revealed TH+ cells and processes within Cl 17 and MAN (Fig. 2F), as well as TH+ processes running in the VNC (Fig. 2G).

### Biochemical Detection Supports the Presence of a TH Immunoreactive Protein in the Brain

**Fig. 3:**
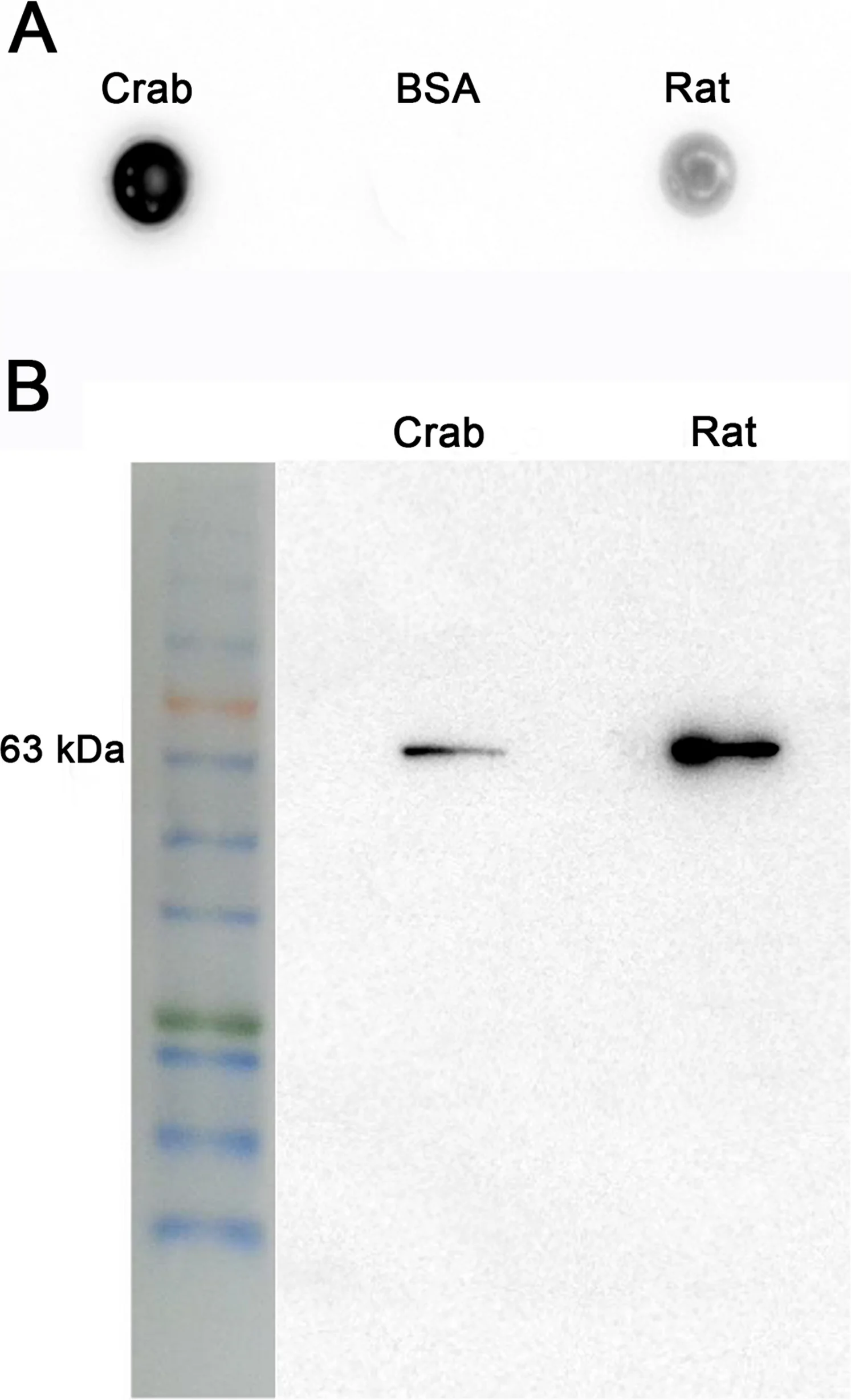
Biochemical detection of tyrosine hydroxylase (TH) in the brain of the crab *Ucides cordatus* and of rats. **A** Dot blot revealing TH in crab brain tissue; rat brain was used as positive control and bovine serum albumin (BSA) as negative control. **B** Western blotting showing immunoreactive bands at approximately 63 kDa in both crab and rat samples, consistent with the expected molecular weight of TH, in kilodaltons (kDa), compared to a molecular weight standard. We used *n* = 4 (pools of 15 crab brains)

To investigate the presence of TH in brain tissue, we performed immunoblot and Western blotting analyses for TH (Fig. 3). The immunoblot (dot blot) supported the presence of a TH-immunoreactive protein (Fig. 3A), whereas Western blotting revealed a single band at approximately 63 kDa, corresponding to the expected molecular weight of TH, in both the *U. cordatus* brain and the rat brain, used as a positive control (Fig. 3B).

### 6-OHDA Induces Progressive Reduction of TH + Cells in Cultured Crab Brain Tissue

**Fig. 4:**
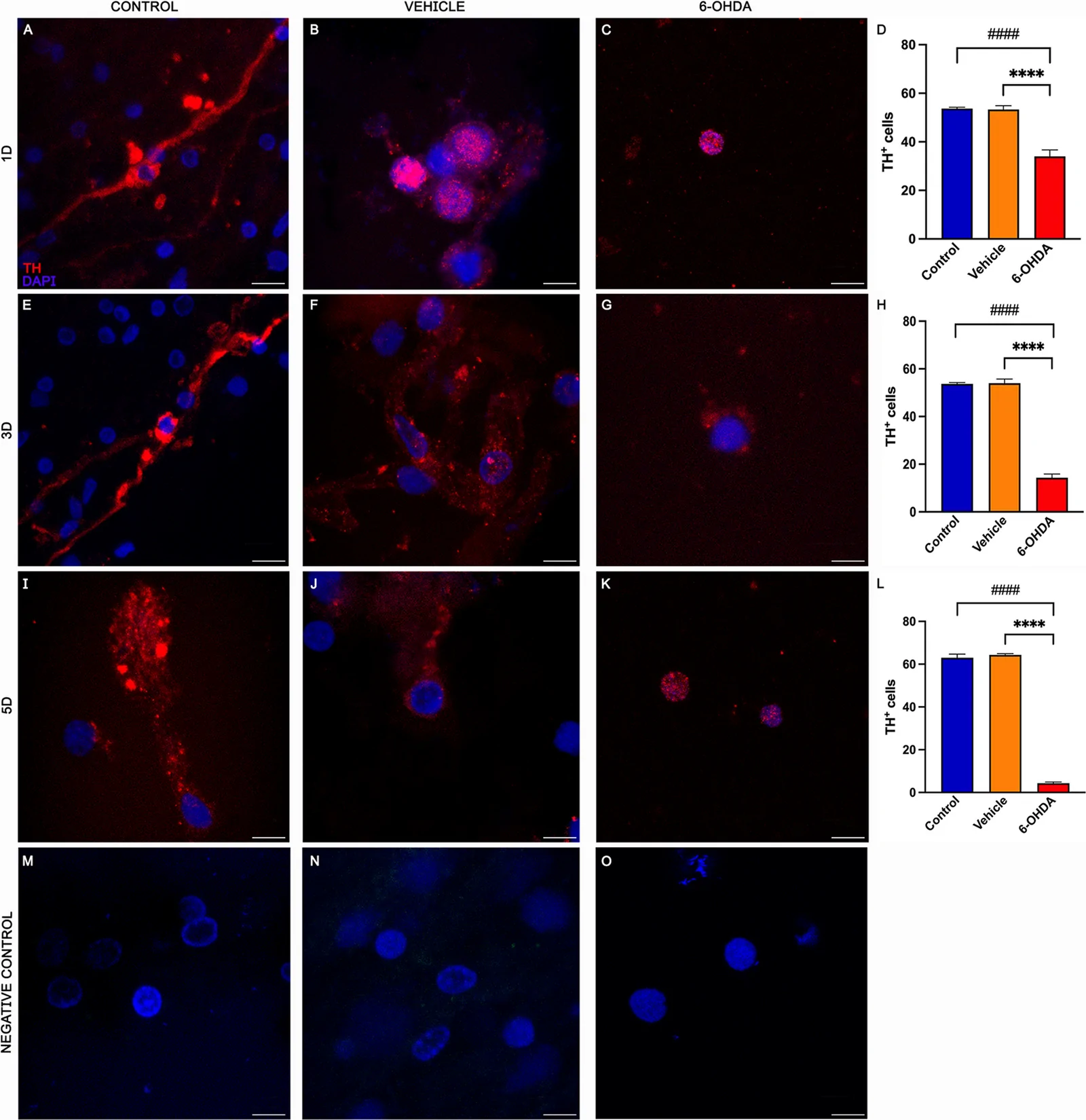
Immunocytochemistry for TH in the brain of the crab under control conditions and after vehicle and 6-OHDA exposure (*n* = 3 of 20 crab brains each day of each group, therefore totaling *n* = 180 crab brains). **A** TH+ cells and their processes in the control group after 1 day in the culture medium. **B** TH+ cells and their processes in the vehicle group after 1 day in the culture medium. **C** TH+ cell after 1 day of exposure to the neurotoxin 6-hydroxydopamine (6-OHDA), showing loss of processes and perinuclear cytoplasm retraction. **D** Quantification of TH+ cells after 1 day of 6-OHDA exposure compared with the control group and the vehicle group. There were fewer TH+ cells in the 6-OHDA (control was 53.67 ± 0.33, vehicle was 53.33 ± 0.88, and 6-OHDA was 34 ± 1.53 TH+ cells). **E** TH+ cells in the control group after 3 days in the culture medium. **F** TH+ cells and their processes in the vehicle group after 3 days in the culture medium. **G** TH+ cell after 3 days of 6-OHDA exposure. **H** Quantification of TH + cells after 3 days of 6-OHDA exposure (control was 53.67 ± 0.33, vehicle was 54 ± 1.0, and 6-OHDA was 14.33 ± 0.88 TH+ cells). **I** TH+ cells in the control group after 5 days in the culture medium. **J** TH+ cells and their processes in the vehicle group after 5 days in the culture medium. **K** TH+ cells after 5 days of 6-OHDA exposure, showing similar morphology to that observed in the other control groups (1 and 3 days), with reduced processes and smaller amounts of cytoplasm. **L** Quantification of TH + cells after 5 days of 6-OHDA exposure compared with the control group (control was 63.0 ± 1.0, vehicle was 64.33 ± 0.33, and 6-OHDA was 4.33 ± 0.33 TH+ cells). **M**–**O** Negative control of the reactions (control and experimental groups, respectively). Scale bars: **A**, **E** (10 µm); **B**, **C**, **F**, **G**, **I**–**K**, **M**–**O** (30 µm). All results are expressed as mean ± standard error (*n* = 3 for each group). ^#^ indicates a significant difference between the control group and 6-OHDA, and ^*^ indicates a significant difference between the vehicle group and 6-OHDA. A one-way ANOVA test followed by Bonferroni’s multiple comparison posttest was performed, ^####^*p* < 0.0001 and ^****^p < 0.0001. Significant differences among groups were observed on day 1 (*F*_2,6_ = 118.0, *p* < 0.0001), day 3 (*F*_2,6_ = 826.0, *p* < 0.0001), and day 5 (*F*_2,6_ = 2881, *p* < 0.0001)

Exposure of primary cultured crab brain tissue to 6-OHDA for 1, 3, and 5 days resulted in marked morphological alterations and reduced numbers of TH+ cells (Fig. 4). Exposed cells showed cytoplasmic retraction toward the perinuclear region, along with significant shortening or loss of neuronal processes, indicating neurotoxin-induced degeneration (Fig. 4A and C, E and G, and I and K). Notably, TH+ cells cultured for 3 and 5 days (Fig. 4E and I) displayed a morphology similar to that of control cells cultured for only 1 day (Fig. 4A). In addition, Fig. 4I shows a cell with an apparent growth cone at 5 days.

Quantitative analysis revealed a significant reduction in the number of TH + cells in the 6-OHDA-administered group compared with both the control and vehicle groups at all time points analyzed: day 1 (control was 53.67 ± 0.33, vehicle was 53.33 ± 0.88, and 6-OHDA was 34 ± 1.53 TH + cells) (Fig. 4D), day 3 (control was 53.67 ± 0.33, vehicle was 54 ± 1.0, and 6-OHDA was 14.33 ± 0.88 TH + cells) (Fig. 4H), and day 5 (control was 63.0 ± 1.0, vehicle was 64.33 ± 0.33, and 6-OHDA was 4.33 ± 0.33 TH + cells) (Fig. 4L). No significant differences were observed between the control and vehicle groups at any time point (Fig. 4D, H, and L).

### DA + Neurons and Projections are Present Across Multiple Deutocerebral Regions

We also conducted immunohistochemical reactions for DA in the brain of the crab *U. cordatus.* We observed DA+ cells and processes in several regions of the deutocerebrum, such as Cl 6, Cl 9, Cl 10, and Cl 17, in addition to AMPN, central body (CB), and MAN (Supplementary Figure).

### TH Sequence Analysis Reveals a Conserved Fragment N-terminal Region Across Species

**Fig. 5:**
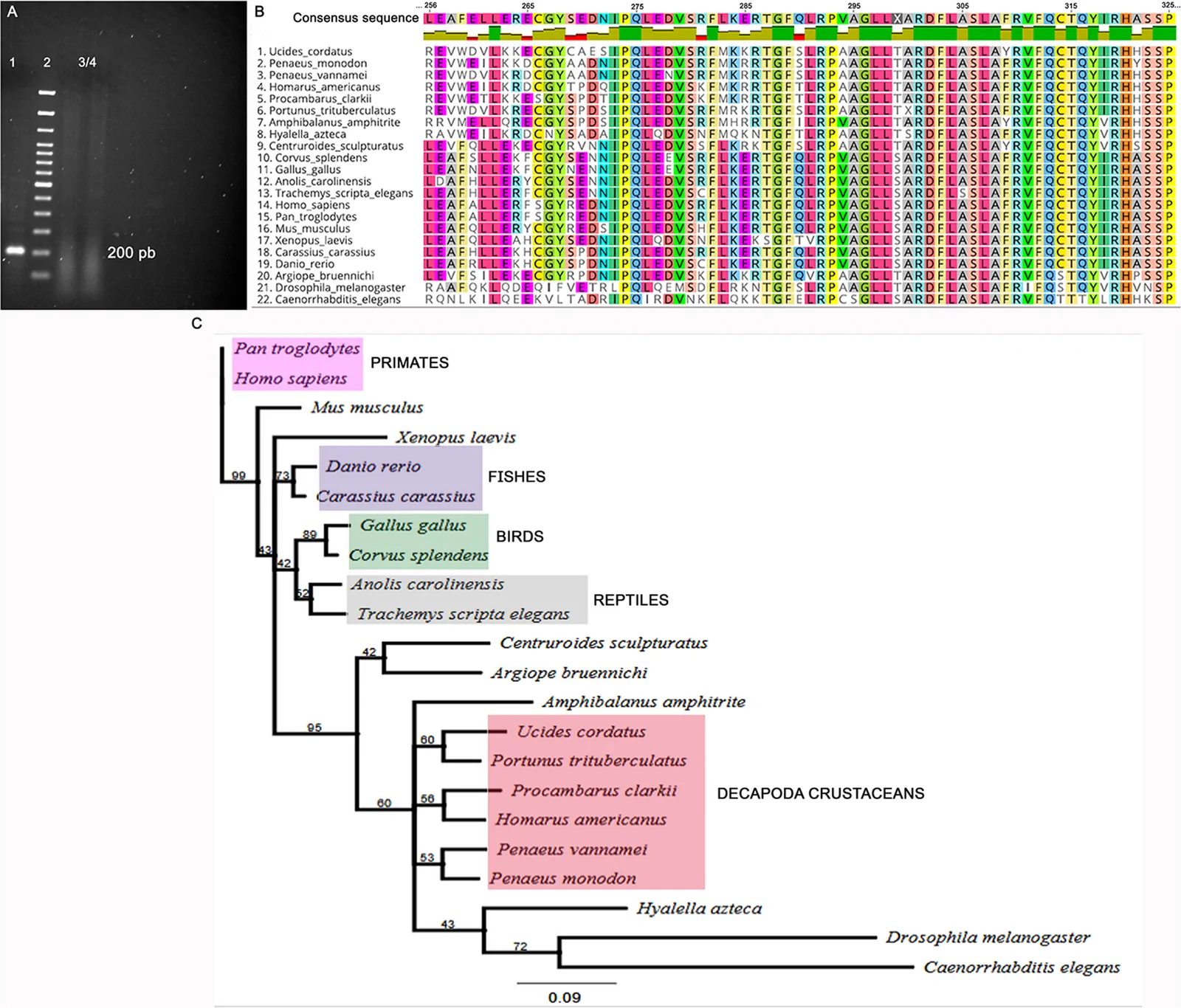
Amplification and analysis of tyrosine hydroxylase (TH) partial coding region (partial cds) of *Ucides cordatus* (five crab brains). **A** Agarose gel electrophoresis of the product of PCR of the cDNA. Lane 1: crab brain *Ucides cordatus* cDNA sample; lane 2: molecular weight pattern; lanes 3 and 4: H_2_O nuclease-free. **B** Partial TH amino acid sequence of *U. cordatus* (access number PZ917597) aligned to TH partial protein sequences of 21 other species. *Homo sapiens* (access number KAI4069453.1), *Pan troglodytes* (chimpanzee; access number XP_016775674.1), *Anolis carolinensis* (green anole; access number XP_062823714.1), *Trachemys scripta elegans* (red-eared slider turtle; access number XP_034626169.1), *Mus musculus* (house mouse; access number AAA40434.1), *Xenopus laevis* (African clawed frog; access number AAR23931.1), *Corvus splendens* (house crow; access number QOW95819.1), *Gallus gallus* (chicken; access number CAB62388.1), *Centruroides sculpturatus* (bark scorpion; access number XP_023240076.1), *Argiope bruennichi* (wasp spider; access number XP_055935929.1), *Amphibalanus amphitrite* (striped barnacle; access number XP_043230680.1), *Hyalella azteca* (amphipod; access number XP_018020964.1), *Drosophila melanogaster* (fruit fly; access number CAA53802.1), *Caenorhabditis elegans* (nematode; access number CDG24121.1), *Portunus trituberculatus* (Asian blue crab; access number XP_045138592.1), *Penaeus monodon* (giant tiger prawn; access number XP_037788831.1), *Penaeus vannamei* (whiteleg shrimp; access number ANA78296.1), *Homarus americanus* (American lobster; access number XP_042215467.1), *Procambarus clarkii* (red swamp crayfish; access number XP_045604547.1), *Carassius carassius* (crucian carp; access number XP_059402605.1), and *Danio rerio* (zebrafish; access number AAI63639.1). Consensus sequence (top line in green). Amino acids are displayed in different colors. Equal colors indicate the same amino acids. **C** A phylogenetic tree derived from the alignment of 21 TH partial protein sequence was constructed using the neighbor-joining method. Numbers in nodes are the bootstrap values. The reliability of the inferred phylogenetic relationships was assessed by bootstrap analysis with 1000 replicates. Bootstrap support values ≥ 50% are displayed on the tree

To elucidate the primary sequence of TH present in the crab brain, we sequenced a 200-bp fragment of crab TH cDNA (GenBank access number PZ917597; Fig. 5A). Translation of this sequence revealed amino acids in the N-terminal region of the enzyme, corresponding to residues 256 to 325, located near the enzyme’s active site [9]. Figure 5B shows the sequence alignment between the crab TH fragment and the same fragment from 21 other species, revealing the presence of shared amino acids, indicative of conservation. Phylogenetic analysis further showed that this fragment is more similar among crab species, e.g., *U. cordatus* and *Portunus trituberculatus* displayed high similarity of amino acid sequences (Fig. 5C). Primates, including chimpanzees and humans, also showed conserved amino acid residues, as did other groups such as birds, fish, and decapod crustaceans.

**Fig. 6:**
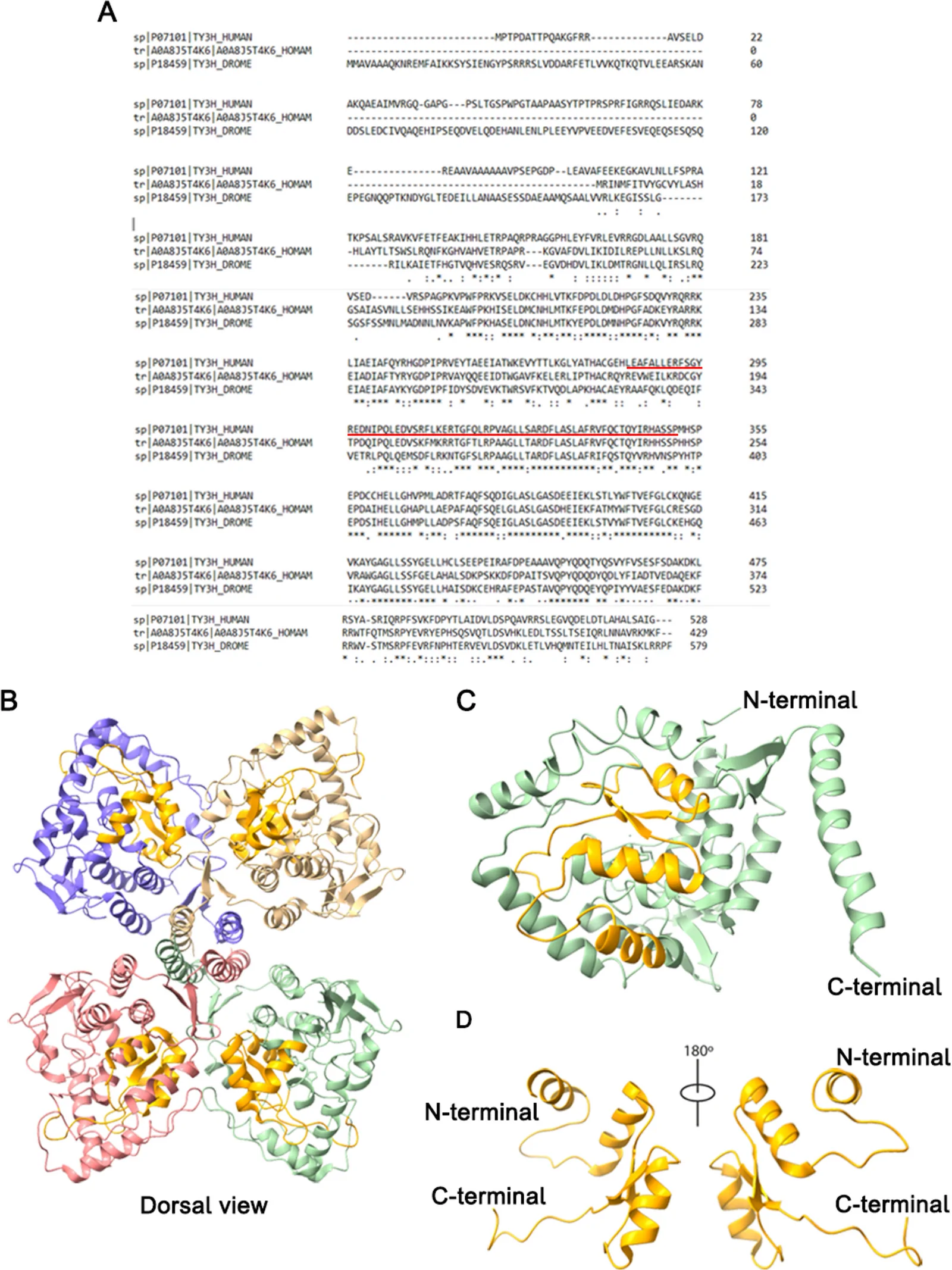
Predicted primary structure of the peptide identified in *Ucides cordatus* and comparison with the human enzyme. **A** Sequence alignment of TH from *Homo sapiens* (HUMAN), *Homarus americanus* (HOMAM), and *Drosophila melanogaster* (DROME) with *U. cordatus*. Asterisks indicate conserved amino acids, and the red line highlights the region selected for three-dimensional structural analysis. **B** Structural superposition of the *U. cordatus* TH region (yellow) onto the human TH homotetramer. **C** Superposition of the *U. cordatus* sequence onto a single monomer of human TH. **D** Ventral and dorsal views of the predicted tertiary structure of the sequenced TH region from *U. cordatus*

The primary sequence of the TH region identified by RNA-seq was aligned and compared with the corresponding TH fragment sequences from *Drosophila melanogaster*, a hexapod species belonging to the sister group of crustaceans; *Homarus americanus*, a species from the same subphylum as our animal model; and *Homo sapiens*. The alignment showed that this region is conserved across species (Fig. 6A). The corresponding region in human TH is highlighted in red.

Next, the structure of human TH was used to compare the identified region in *U. cordatus* with its human counterpart. Under homeostatic conditions, human TH adopts a tetrameric structure (Fig. 6B). The region identified in *U. cordatus* is highlighted in yellow in both the tetrameric (Fig. 6B) and monomeric (Fig. 6C) conformers. We modeled the tertiary structure of the corresponding *U. cordatus* fragment sequence (69-mer peptide). Notably, the predicted structure of this fragment closely resembles the analogous region in human TH, reinforcing the notion that this region is structurally conserved between *H. sapiens* and *U. cordatus*.

## Discussion

The present study demonstrated that TH is widely distributed in the brain of *U. cordatus*, supporting the existence of an extensive catecholaminergic system in this species. TH immunoreactivity was detected in multiple brain regions, including Cl 6, Cl 9, Cl 10, Cl 17, MAN, OL, and VNC, indicating that dopaminergic neurons are broadly integrated within sensory and integrative centers of the crab nervous system. In addition to identifying brain regions containing TH and DA, we showed that the molecular weight of *U. cordatus* TH is consistent with that of vertebrate TH. We also characterized a fragment of TH primary sequence and predicted the molecular structure of this fragment through RNA sequencing and AlphaFold modeling, providing insights into its tertiary organization. Finally, we performed a phylogenetic analysis of the phylogenetic similarity of the TH fragment among a range of invertebrates and vertebrates.

### Dopaminergic Circuits Were Found in Motor and Sensory Brain Areas

The labeling of Cl 6 may be related to the classic dopaminergic function of motor control, since this region is canonically associated with motor activity in other crustacean species [20, 25]. The study by [20] showed degeneration and consequent lower activation of this region when the animals’ legs underwent induced or natural autotomy. It was described a possible anatomical pathway related to movement control and neuronal modulation [28] in a crab model. This study [28] showed that cells in Cl 6 project to and connect with AMPN neurons. These findings and the histological results of our study suggest that dopaminergic cells from Cl 6 project to AMPN and may be related to motor and neuromodulator functions. The same study [28], together with the results from [25, 33, 34], found that AMPN cells project to the CB. In turn, the CB projects to two distinct locations: Cl 9, where the actions of local interneurons are modulated, and MAN, responsible for processing sensory information captured by the antenna. Similarly, we found DA in several cells in Cl 9 and MAN, which suggests that dopaminergic cells may modulate the circuitry of neurotransmitters in these regions. Additionally, [28] also showed that MAN projects to Cl 17 and to VNC, responsible for the control of the animal’s legs. We also observed dopaminergic neurons in these neuropils; therefore, DA may be used as a modulator for other neurotransmitters or, alternatively, as a neurotransmitter that controls the movement of the legs. We also observed DA labeling in Cl 10, and other studies [33, 35, 36] confirm that this cluster has neurons that project to the eyestalk, controlling sensory inputs in a negative feedback mechanism.

We suggest that TH + cells in the OL are associated with the final stages of sensory processing and the development of motor responses, as described in lobster, crayfish, and other crab models [19, 20, 35]. Notably, the presence of TH in the OL, a region involved in olfactory input processing, suggests that this molecule plays an important role in olfaction. Indeed, degeneration of dopaminergic terminals has been associated with impaired olfactory processing, consistent with evidence from vertebrate studies showing loss of dopaminergic neurons [37]. Other studies in vertebrate models have also reported marked reductions in TH and DA in olfactory regions, such as the olfactory bulb. Given the inhibitory role of DA in olfactory processing, these alterations may be related to hyposmia [38, 39]. Moreover, the immunohistochemical detection of DA in regions overlapping with TH distribution—including Cl 6, Cl 9, Cl 10, AMPN, CB, and MAN—indicates that some TH + neurons may be dopaminergic.

### 6-OHDA Reduced the Number of Catecholaminergic Neurons

The observation that cells cultured for 3 and 5 days resembled control cells at earlier stages suggests progressive structural impairment rather than immediate cell death. Moreover, the significant reduction in TH+ cell numbers after 6-OHDA exposure indicates the vulnerability of catecholaminergic neurons, consistent with the known mechanism of this neurotoxin [40, 41], which induces oxidative stress and degeneration in dopaminergic cells.

### Structure of the Sequenced TH Fragment Shows Particular Amino Acid Residues Among Species

Biochemical confirmation by immunoblot and Western blot analyses agreed with the anatomical findings. The detection of a single band at approximately 63 kDa, corresponding to the expected molecular weight of the vertebrate TH, indicates that the immunohistochemical reaction was labeling this enzyme in crab brain tissue. PCR amplification using primers designed from *Litopenaeus vannamei* also supported the Western blot results, consistent with the conservation of this TH fragment in this species relative to vertebrates and invertebrates [42]. Sanger sequencing revealed conservation of the sequenced region in *U. cordatus*, located near the enzyme’s active site within the N-terminal domain. Across animal evolution, tyrosine has been maintained as the substrate of TH, indicating that amino acid–substrate interactions at the active site, as well as the presence of specific residues in its vicinity, were conserved over time [13, 42, 43]. Together, these findings reinforced the evolutionary conservation of the sequenced fragment of *U. cordatus* TH, indicating that the role of this fragment is maintained across species.

Cysteine residues at positions 266 and 314 may contribute to the stabilization of the three-dimensional structure of TH, given that the mammalian enzyme contains seven cysteine residues, with only slight variation among species [9, 44]. Studies on mammalian TH have shown that cysteine residues are important for the organization and stabilization of its three-dimensional structure [13]. The presence of these cysteine residues in the brain of our animal model, similarly to some other arthropods, might possibly confer additional stability of the molecule.

In vertebrate models, the AGLL sequence (alanine–glycine–leucine–leucine) at positions 295–298 has been reported to reduce TH flexibility. This motif modulates enzyme dynamics during substrate binding, impairing enzyme saturation and reducing the reaction rate [8, 9]. Our results revealed the same amino acids at the corresponding positions, suggesting that these residues may play a similar functional role in our model. In addition, the RDFL sequence (arginine–aspartate–phenylalanine–leucine) at positions 301–304, also present in our model, is important for binding the coenzyme tetrahydrobiopterin (BH4), in both vertebrates and invertebrates [8, 9, 42]. In this region, phenylalanine directly interacts with BH4 and is supported by the surrounding residues arginine, aspartate, and leucine [45]. Here, we hypothesize that the coenzyme may link to these same residues.

Serine and phenylalanine residues at positions 280 and 282 have been found to participate in tyrosine binding in both vertebrate and invertebrate models [9, 42]. Our analysis revealed the same residues at the corresponding positions, suggesting that they may be important for substrate interaction across species. Moreover, the SSP motif (serine–serine–proline) at positions 323–325, also identified in our model, may help to stabilize the conformation of the catalytic site [10].

### Evolutionary and Environmental Adaptations of TH

The predicted structure of the TH fragment indicated that the sequenced region is preserved, as most parts of the fragment overlapped with the corresponding region of human TH. The overlapping of alpha-helix and beta-sheet suggests that interactions with the coenzyme BH4, the substrate, and other molecules may be structurally preserved across species. Differences were also observed, some regions lacking overlap between crab and human proteins. These discrepancies may reflect amino acid substitutions between species, which changes in the intertwining and orientation of the enzyme.

The phylogenetic similarity analysis of this fragment showed that primates, chimpanzees, and humans share identical amino acids, and greater similarity was observed between sister organisms, such as birds, fish, and decapod crustaceans. Greater similarity of this fragment was also identified between crab species, since *U. cordatus* and *Portunus trituberculatus* showed similarity between the amino acid sequences. These findings suggest that the adaptability of the substrate interaction mediated by this region of the active site remained largely conserved across the species analyzed [43]. Additionally, the conservation of this fragment of TH provides information that may be useful for future studies related to some aspects of parkinsonism in crustaceans.

## Conclusion

Taken together, our results revealed a TH fragment structurally conserved across 21 vertebrate and invertebrate species. The results also demonstrated a complex dopaminergic network in *U. cordatus*, characterized by diverse neuronal morphologies, widespread projections, and sensitivity to dopaminergic neurotoxins. This organization suggests that DA may regulate sensory processing, neural integration, and motor control in crustaceans. Therefore, these findings support the use of this species as a comparative model for studying catecholaminergic neurobiology. This model may be useful for further studies on neurodegenerative conditions.

## Supplementary Information

ESM 1 Immunohistochemistry for dopamine (DA) in the brain of the crab Ucides cordatus (n = 5). (A, D, G) DA + cells in the right side (R) of the deutocerebrum. (B, E, H) DA + cell bodies (arrows) in the medial (M) deutocerebrum and in the central body (CB). (C, I) DA + cell bodies (arrows) in the left side (L) of the deutocerebrum. (F) The dotted square shows a few DA + cell bodies. (A, B, C) Processes (arrowheads) are labeled within cluster 6 (Cl 6) and anterior medial protocerebral neuropils (AMPN). (D, F) DA + processes (arrowheads) are labeled within cluster 9 (Cl 9). The insert in F is a DA + cell group. (G, H, I) Processes (arrowheads) are labeled within cluster 10 (Cl 10) and medial anterior neuropil (MAN). Arrows indicate DA + cell bodies. The insert in I is the negative control of the reaction. Scale bars: A–D (42 µm), E and insert (17 µm), F–H (42 µm), and I (60 µm) (PNG 2.13 MB)

High resolution image (TIF 9.07 MB)

ESM 2 (PNG 116 KB)

High resolution image (TIF 1.83 MB)

ESM 3 (PNG 283 KB)

High resolution image (TIF 4.03 MB)

## Data Availability

No datasets were generated or analyzed during the current study.

## Declarations

### Declaration of AI Use

We have not used AI-assisted technologies in creating this article.
